# Stress analysis under centrifugal load of the multiple corrugated diaphragm coupling based on rotating shell thin film model

**DOI:** 10.1038/s41598-024-60933-7

**Published:** 2024-05-02

**Authors:** Angang Cao, Shiru Sun

**Affiliations:** 1 School of Mechanical Engineering, Zhengzhou University of Science and Technology, Zhengzhou, 450064 China; 2 School of Material Science and Engineering, Henan Polytechnic University, Jiaozuo, 454000 China

**Keywords:** Rotating shell thin film mode, Centrifugal load, Multiple corrugated diaphragm, Analytical method, Finite element method, Energy science and technology, Engineering

## Abstract

Centrifugal force is one of the factors that cannot be ignored in high-speed shaft systems. The multiple corrugated diaphragm (MCD) Coupling is suitable for high-power and high-speed situations; hence, it is crucial to investigate the stress and deformation of the wave disc under centrifugal load. This paper first uses the rotating shell thin film model to derive the circumferential stress and deformation displacement of the MCD under centrifugal load. Then, the finite element method is used to verify the results obtained from the analytical solution. The results show the feasibility of using the rotating shell thin film model for centrifugal load analysis of the MCD, providing a new approach for the theoretical analysis of the MCD.

## Introduction

The multiple corrugated diaphragm (MCD) coupling is a new type of flexible coupling developed based on the diaphragm coupling and the disc coupling, with its core components being a group of high-strength and high-toughness stainless steel discs. The multiple separated diaphragm within the diaphragm group has inherent fault protection functions, increasing the coupling’s flexibility and obtaining greater torque transmission capacity without increasing the diameter of the coupling. The profile of the mutually parallel corrugated diaphragm is increased by unfolding the waveform when transmitting power and compensating for deformation. Therefore, compared with the traditional profile diaphragm coupling, the MCD coupling has advantages such as a high torque-to-diameter ratio, a high torque-to-weight ratio, and a large compensation capacity. MCD suits high-power, high-speed operating conditions^1–5^. For high-speed shaft systems, centrifugal force is one of the factors that cannot be ignored. Therefore, it is crucial to investigate the stress and deformation of the MCD under centrifugal load^6^.

Several investigations on the coupling applied in different fields were conducted. Buryy et al.^7^ proposed a calculation method for the torsional stiffness of the flexible disc coupling based on the study of the finite element model response of the flexible disc coupling under the action of torque. The sufficiency of the calculation results and the model was confirmed through experimental measurements, which is enough to meet practical applications. Xia et al.^8^ established a six-degree-of-freedom dynamic model comprising two rigid rotors using the Lagrange method. The rigid rotor is connected by a hexagonal, flexible coupling. The research results provide theoretical support for the fault diagnosis and detection of rotating machinery connected by hexagonal flexible couplings. Li et al.^9^ took a new type of coupling as the research object. They used the finite element method and the lumped parameter method to analyze the axial vibration mode of the coupling. The authors analyzed the influence of axial expansion and speed on the axial critical speed of the diaphragm coupling. Mancuso^10^ introduced several couplings used for gas turbines and mentioned that the corrugated diaphragm coupling is more suitable for supporting gas turbines. However, the article did not introduce the design calculation method of the corrugated diaphragm. Duong and Kazerounian^11^ conducted an in-depth study of commercial mechanical coupling diaphragms. The authors showed that the structural performance of a flexible tapered diaphragm coupling is mainly associated with the stress intensity and stress trajectories at the junction of the diaphragms ‘outer diameter to the rim support structure. Junfeng et al.^12^ calculated the diaphragm coupling stiffness through the empirical formula method, the single diaphragm linear superposition model, and the method of the diaphragm group. The authors provided an important reference for the simulation calculation of the stiffness of the diaphragm coupling. Liu et al.^13^ used Workbench software to establish a finite element model of the diaphragm coupling. They analyzed the impact of the diaphragm structure on the torsional stiffness of the coupling. The authors provided significant results for guiding the design of the diaphragm coupling. Wang et al.^14^ used the finite element software ABAQUS to establish a finite element model of the large rubber block diaphragm coupling. Moreover, they calculated the static torsional stiffness and radial and axial stiffness of the diaphragm rubber block coupling, guiding further design research. Ai et al.^15^ used the finite element analysis software ANSYS and the secondary development language APDL to conduct research and analysis on the characteristics of the metal flexible diaphragm coupling. Li^16^ researched the corrugated diaphragm’s structural strength, fatigue life, and vibration characteristics by establishing a parametric finite element model.

Most research on couplings is based on experimental testing or finite element methods, with relatively less research on theoretical derivation and even less on MCD couplings. The approximate solutions obtained using finite element methods cannot help deeply analyze this special waveform structure. Therefore, it is necessary to conduct theoretical research on the MCD to promote the application and development of the MCD couplings.

This paper uses the rotating shell thin film model to derive the circumferential stress and deformation displacement of the MCD under centrifugal load. Then, the finite element method is used to verify the results obtained by the analytical solution. The results show the feasibility of using the rotating shell thin film model to analyze the centrifugal load of the MCD coupling^17,18^.

## Materials and methods

### Parameters of the MCD

**Figure 1:**
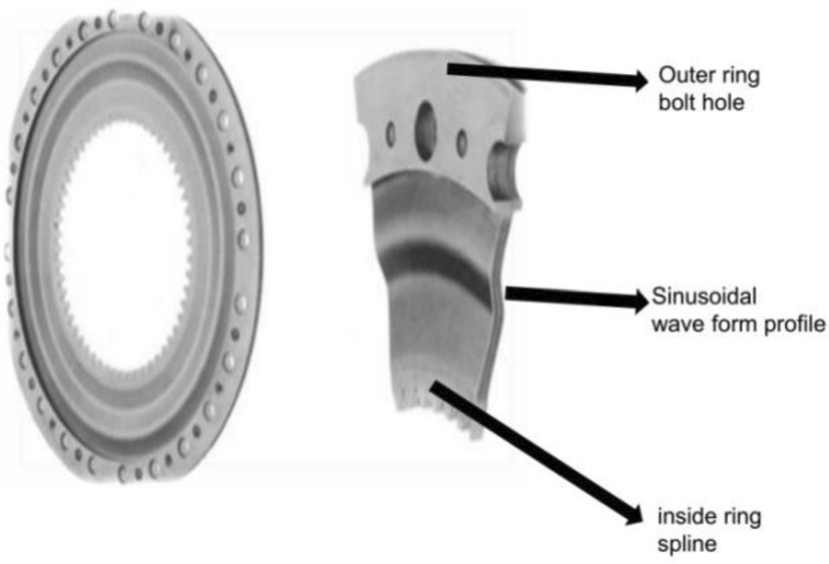
MCD shape.

The shape of the MCD is shown in Fig. 1. The specific geometric and working condition parameters of the MCD in this paper are shown in Table 1. The subsequent analysis process neglects the influence of bolt holes and spline keys.

**Table 1:** Geometric parameters of the MCD.

Serial number	Parameter	Symbol/unit	Value
1	The inner radius of the profile	b/mm	104
2	The outside radius of the profile	a/mm	162
3	The thickness of the profile	h/mm	0.5
4	The amplitude of the profile	H/mm	3.5
5	The thickness of the hub	h1/mm	1.2
6	The thickness of the flange	h2/mm	1.2
7	Transition Fillet Radius at the Hub	r2/mm	2
8	Transition Fillet Radius at the Rim	r3/mm	2
9	The radius of the inner circle	r0/mm	89
10	The radius of the outer circle	r1/mm	182
11	The number of diaphragms in an assembly	m/piece	15
12	Operating speed (working condition parameter)	r/min	5200

### Circumferential force of the MCD under centrifugal load

Since centrifugal force is an axisymmetric volume load, the MCD under centrifugal action can be regarded as a rotating shell thin film model under the action of axisymmetric volume load. First, the rotating shell thin film model's force balance (differential) equation is derived. Then, the centrifugal loading condition is introduced to find the meridional and latitudinal tension of the rotating shell. Since the structure of the MCD is quite special, its axial section is a complete period of a sine curve. Multiple singular points will appear due to the sudden change in the radius of curvature in the sine curve and two extreme points on the curve. The axial section is analyzed and calculated in the Cartesian coordinate system to avoid the occurrence of singular points and the influence of the radius of curvature.

**Figure 2:**
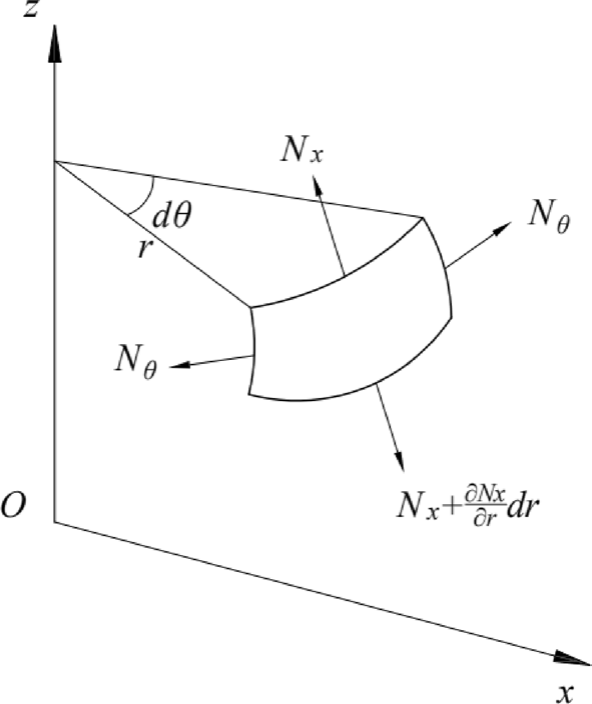
Division of diaphragm elements.

A rotating shell is formed by a plane curve rotating about an axis coplanar with this curve. This curve is called a meridian, and the plane where the curve is located is called the meridian plane. The horizontal circumference formed by any point on the curve rotating about the axis is called a parallel circle. Figure 2 shows a differential element cut from the rotating shell's middle surface using two adjacent meridians and two adjacent parallel circles.

Because both the meridians and parallel circles are curvature lines of the rotating surface, these two families of curves are taken as coordinate lines. The position of the meridian is determined by the angle $\theta$ measured from a certain reference meridian plane. In contrast, the position of the parallel circle is established in the Cartesian coordinate system with the rotation axis as the z-axis and the curve extension direction as the x-axis. Here, r is the curvature radius of the parallel circle.

The coordinate axes are defined as follows. The z-axis is perpendicular to the parallel circle, the x-axis is parallel to the circle, and the *y*-axis is perpendicular to the *XOZ* plane. The direction of the coordinate axes is shown in Fig. 2. The external force acting on each unit area of the shell surface has components $p_{x}$, $p_{y}$, and $p_{z}$ along the positive direction of the coordinate axes.

Next, the balance of the rotating shell element will be investigated (Fig. 2). Three balance equations will be provided based on the balance conditions of the forces in the direction of the three coordinate axes. These three balance equations determine the unknown internal forces $N_{x}$, $N_{\theta }$.

First, the tangential force on the infinitesimal body is analyzed:

Since the centrifugal load is axisymmetric, the infinitesimal body is not subjected to shear force; the diaphragm undergoes axisymmetric deformation under the action of the load. Therefore, the circumferential force $N_{\theta }$ does not change with $\theta$, i.e.:

1 $$ \frac{{\partial N_{\theta } }}{\partial \theta } = 0. $$

**Figure 3:**
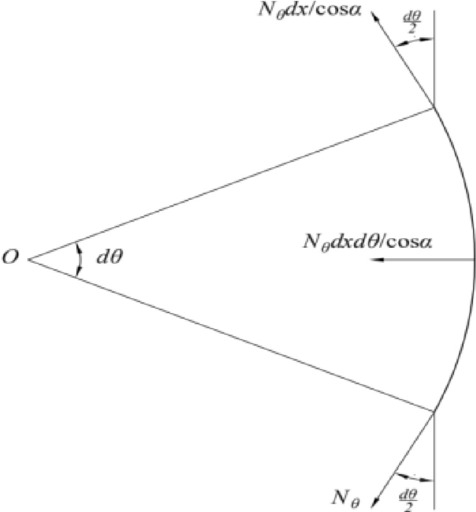
Circumferential force analysis diagram of a microelement.

The analysis of the circumferential force on the infinitesimal is shown in Fig. 3. Since the latitude line is parallel to the radius of curvature of the circle, *r*, the arc length of the infinitesimal meridian is:

2 $$ ds1 = rd\theta , $$

3 $$ r = x. $$

The arc length is simplified to a straight line in the cross-sectional diagram of the diaphragm. Therefore, the length of the straight line is:

4 $$ ds = \frac{r}{\cos \alpha }. $$

The element area is:

5 $$ S = \frac{r}{\cos \alpha }d\theta dx. $$

The force exerted along the latitude line in the direction parallel to the circle:

6 $$ \frac{{N_{\theta } }}{\cos \alpha }dx. $$

The angle between the circumferential force and the vertical direction is $d\theta /2$. The two forces are projected onto the *x*-axis pointing to *O* point:

7 $$ 2\left( {\frac{{N_{\theta } dx}}{\cos \alpha }} \right)\sin \frac{d\theta }{2}. $$

Since $d\theta$ is very small:

8 $$ \sin \frac{d\theta }{2} \approx \frac{d\theta }{2}. $$

The sum of the two forces simplifies to:

9 $$ \frac{{N_{\theta } }}{\cos \alpha }dxd\theta . $$

**Figure 4:**
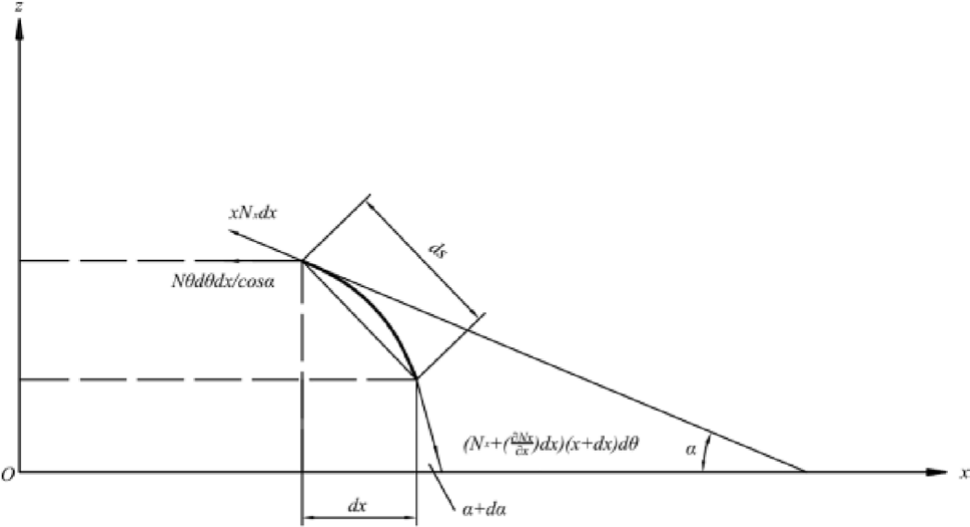
Circumferential force analysis diagram of microelement.

The tangential force on the cross-section within the *xoz* plane in the infinitesimal tangential force analysis diagram is shown in Fig. 4. Since the circular arc is equivalent to a straight line, the tangential force is oriented along the direction of the straight line, where the negative direction is:

10 $$ N_{x} xd\theta . $$

The force along the positive direction is:

11 $$ (N_{x} + \frac{{\partial N_{x} }}{\partial x}dx)(x + dx)d\theta . $$

A balance equation in the tangential direction can then be established:

12 $$ (N_{x} + \frac{{\partial N_{x} }}{\partial x}dx)(x + dx)d\theta - xN_{x} d\theta - N_{\theta } dxd\theta + p_{y} \frac{r}{\cos \alpha }d\theta dx = 0. $$

The third-order small quantity of the equation is neglected to obtain:

13 $$ N_{x} - N_{\theta } + x\frac{{\partial N_{x} }}{\partial x} + \frac{{p_{y} x}}{\cos \alpha } = 0. $$

Considering the normal force, the tangential and circumferential forces are projected onto the normal line to obtain:

14 $$ N_{\theta } dxd\theta \tan \alpha - p_{z} xd\theta \frac{dx}{{\cos \alpha }} = 0. $$

After simplification:

15 $$ N_{\theta } \sin \alpha dxd\theta - p_{z} xd\theta dx = 0. $$

Therefore, the radial balance equation can be written as:

16 $$ N_{\theta } = \frac{{p_{z} x}}{\sin \alpha }. $$

**Figure 5:**
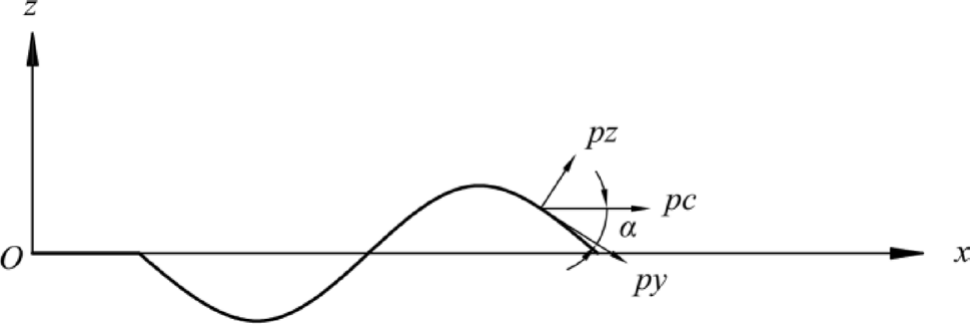
Load diagram.

The load situation is shown in Fig. 5. The centrifugal load acting on the shell element can be regarded as the product of the distributed load intensity $p_{c}$ and the infinitesimal area S. This load intensity $p_{c}$ can be decomposed into $p_{z}$ and $p_{y}$ on the curve's normal and tangential directions. Without considering the thickness of the diaphragm, the centrifugal force is $\rho S\omega^{2} x$, the centrifugal load per unit area is $\rho \omega^{2} x$, the density of the rotating shell is $\rho$, and the angular velocity of the diaphragm is $\omega$. Then, the relationship formula for the centrifugal load is:

17 $$ p_{c} = \rho \omega^{2} x, $$

18 $$ p_{z} = \rho \omega^{2} x\sin \alpha , $$

19 $$ p_{y} = \rho \omega^{2} x\cos \alpha , $$

The centrifugal load and the support condition of the shell are axisymmetric. Therefore, the internal force and deformation of the shell must be symmetrical to the rotation axis. Simultaneously, the shear force must be zero; otherwise, the shear force will cause non-axisymmetric deformation. Moreover, other internal stresses do not change with $\theta$; otherwise, the condition of axisymmetry will be violated. The centrifugal load is substituted into Eqs. (13) and (16) to obtain:

20 $$ N_{\theta } = \rho h\omega^{2} x^{2} . $$

The tangential balance equation is:

21 $$ x\frac{{\partial N_{x} }}{\partial x} + N_{x} + \rho \omega^{2} x^{2} - N_{\theta } = 0. $$

Equation (21) is a first-order linear differential equation with constant coefficients; the solution to the differential equation is:

22 $$ N_{x} = \frac{1}{x}[\int {(N_{\theta } - \frac{{p_{y} x}}{\cos \alpha })} dx + C]. $$

Parameter $N_{\theta }$ is substituted into Eq. (20) to obtain:

23 $$ N_{x} = \frac{1}{x}[\int {( - \rho \omega^{2} x^{2} + \rho \omega^{2} x^{2} )} dx + C]{ = }0. $$

Both ends of the straight line model are processed, the impact of $d_{\alpha }$ on the model is considered, and a balance equation is established in the tangential direction:

24 $$ (N_{x} + \frac{{\partial N_{x} }}{\partial x}dx)(x + dx)d\theta - xN_{x} d\theta - N_{\theta } dxd\theta + p_{y} \frac{r}{\cos \alpha }d\theta dx = 0. $$

The third order and higher small quantities of the equation are neglected to obtain:

25 $$ N_{x} - N_{\theta } + x\frac{{\partial N_{x} }}{\partial x} + \frac{{p_{y} x}}{\cos \alpha } = 0. $$

The tangential and circumferential forces are projected onto the radial direction to obtain:

26 $$ N_{\theta } dxd\theta \tan \alpha + N_{x} xd\theta \sin \left( {\frac{d\alpha }{2}} \right) + (N_{x} + \left( {\frac{{\partial N_{x} }}{\partial x}} \right)dx)(x + dx)d\theta \sin \left( {\frac{d\alpha }{2}} \right) - p_{z} xd\theta \frac{dx}{{\cos \alpha }} = 0. $$

The third order and higher small quantities of the equation are neglected to obtain:

27 $$ N_{\theta } + N_{x} \cot \alpha x\frac{d\alpha }{{dx}} - \frac{{p_{z} x}}{\sin \alpha } = 0. $$

Equations (25) and (27) are added to obtain:

28 $$ \frac{{\partial N_{x} }}{\partial x} + \frac{{N_{x} }}{x} + N_{x} \cot \alpha \frac{d\alpha }{{dx}} + \frac{{p_{y} }}{\cos \alpha } - \frac{{p_{z} }}{\sin \alpha } = 0. $$

The centrifugal load relationships

29 $$ p_{z} = \rho h\omega^{2} x\sin \alpha , $$

30 $$ p_{y} = \rho h\omega^{2} x\cos \alpha , $$

are then substituted to obtain

31 $$ \frac{{dN_{x} }}{dx} + \frac{{N_{x} }}{x} + N_{x} \cot \alpha \frac{d\alpha }{{dx}} = 0. $$

According to the cross-section of the diaphragm:

32 $$ y = ab\sin (b(x - c))). $$

The slope of the curve is:

33 $$ \dot{y} = ab^{2} \cos (b(x - c))). $$

On account of:

34 $$ \dot{y} = ab^{2} \cos (b(x - c))) = \tan \alpha . $$

The relationship between radius $r$ and angle $\alpha$ is obtained as:

35 $$ \alpha = \arctan (ab\cos (b(x - c))). $$

Differentiating both sides of the equation with respect to *x* yields:

36 $$ \frac{d\alpha }{{dx}} = - \frac{{ab^{2} \sin (b(x - c))}}{{1 + (ab\cos (b(x - c)))^{2} }}. $$

Substituting the above expression into Eq. (31) yields:

37 $$ \frac{{dN_{x} }}{dx} + N_{x} (\frac{1}{x} - \frac{{\cot \alpha ab^{2} \sin (b(x - c))}}{{1 + (ab\cos (b(x - c)))^{2} }}) = 0. $$

The solution to the differential equation is obtained as:

38 $$ N_{x} = Ce^{{\int {(\frac{b\tan (b(x - c))}{{(1 + (ab\cos (b(x - c)))^{2} )}} - \frac{1}{x})dx} }} . $$

When *x* takes values at the peak and trough points, $e^{{\int {(\frac{b\tan (b(x - c))}{{(1 + (ab\cos (b(x - c)))^{2} )}} - \frac{1}{x})dx} }}$ tends to infinity. The constant *C* = 0 due to the tangential force, i.e. $N_{x} = 0$.

### Displacement of the MCD under centrifugal load

**Figure 6:**
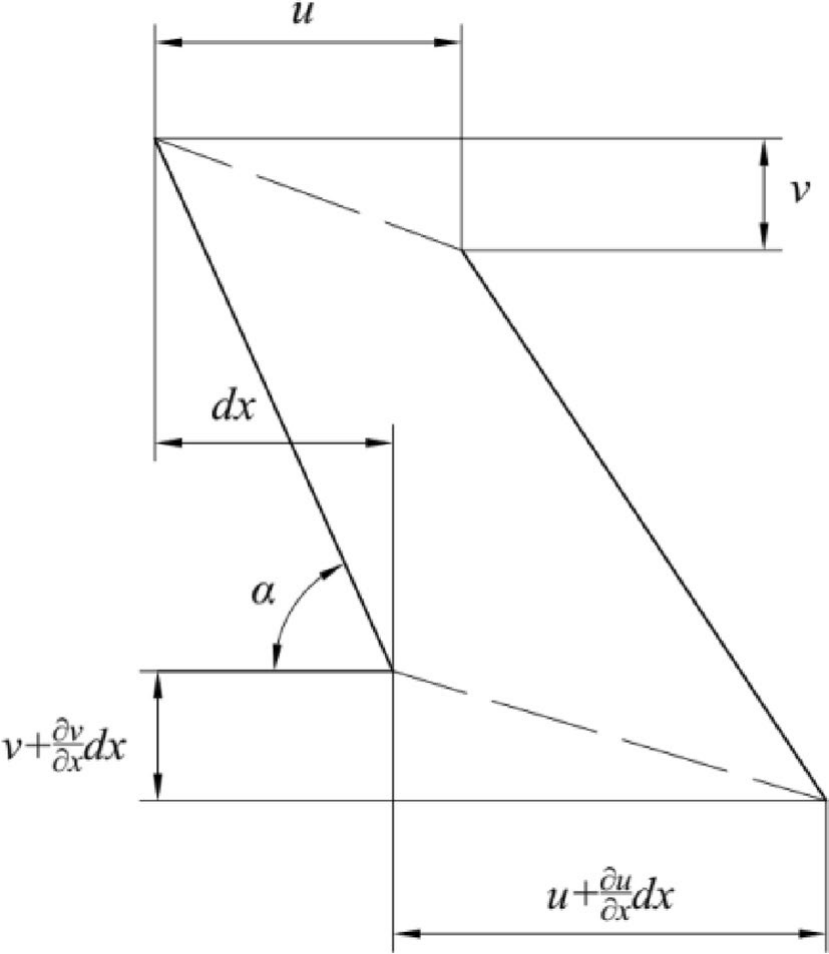
Displacement deformation diagram.

In the case of shell symmetric deformation, a small displacement of a point can be decomposed into two components: displacement *u* along the positive direction of *x*, and displacement *v* along the negative direction of the *z*-axis (as shown in Fig. 6). The arc is simplified to a straight line to eliminate the influence of the curvature radius of the arc^19,20^. The increments of displacements *u* and *v* are $\frac{\partial u}{{\partial x}}$ and $\frac{{\partial {\text{v}}}}{\partial x}$, respectively. Therefore, the total change in the length of its linear infinitesimal unit is:

39 $$ \frac{du}{{\cos \alpha }} + \frac{dv}{{\sin \alpha }}. $$

The increment of straight-line length is decomposed into horizontal and vertical directions. The total change in the straight-line length is divided by the initial length $\frac{dx}{{\cos \alpha }}$ of the unit to obtain the strain in the meridian direction of the inner shell:

40 $$ \varepsilon_{x} = \frac{du}{{dx}} + \frac{dv}{{dx}}\cot \alpha . $$

The displacement deformation for a unit of parallel circles is shown in Fig. 6. The increment of the radius *r* of the circle is *u* due to displacements *v* and *u*. Since the increment of the circumference of the parallel circle is proportional to the increment of the radius, the circumferential strain is:

41 $$ \varepsilon_{\theta } = \frac{u}{x}. $$

According to Hooke’s law, the strains $\varepsilon_{x}$ and $\varepsilon_{\theta }$ are expressed in terms of stresses $N_{x}$ and $N_{\theta }$ as follows:

42 $$ \varepsilon_{\theta } = \frac{{(N_{\theta } - \mu N_{x} )}}{Eh}, $$

43 $$ \varepsilon_{x} = \frac{{(N_{x} - \mu N_{\theta } )}}{Eh}. $$

Hooke’s law is substituted into the circumferential strain $\varepsilon_{\theta }$:

44 $$ u = \frac{x}{Eh}(N_{\theta } - \mu N_{x} ). $$

Diaphragm thickness is introduced since it is related to displacement. The centrifugal load can then be expressed as:

45 $$ p_{c} = \rho h\omega^{2} x. $$

Substituting the expression into stress yields

46 $$ N_{x} = 0, $$

47 $$ N_{\theta } = \rho h\omega^{2} x^{2} . $$

The displacement is obtained as:

48 $$ u = \frac{{\rho \omega^{2} x^{3} }}{E}. $$

Hooke’s law and displacement* u* are substituted into Eq. (40):

49 $$ \frac{dv}{{dx}}\cot \alpha = - \frac{{(\mu + 3)\rho \omega^{2} x^{2} }}{Eh}. $$

The displacement *v* is obtained as follows:

50 $$ v = - \int {(\tan \alpha \frac{{(3 + \mu )\rho \omega^{2} x^{2} }}{{E{\text{h}}}}} )dx + C. $$

The expression $v = - \int {(\tan \alpha \frac{{(3 + \mu )\rho \omega^{2} x^{2} }}{{E{\text{h}}}}} )dx + C$ is substituted into Eq. (50) and integrated:

51 $$ {\text{v}} = - \frac{{(3 + \mu )\rho \omega^{2} }}{E}(ax^{2} sin\left( {b\left( {x - c} \right)} \right) - \frac{{\left( {2asin\left( {b\left( {x - c} \right)} \right) - 2abxcos\left( {b\left( {x - c} \right)} \right)} \right)}}{{b^{2} }}) + C. $$

The outer ring of the corrugated diaphragm is fixed. Therefore, according to the boundary conditions, when x = 162 (Outermost ring of MCD profile), the vertical displacement v = 0. Hence, the constant C can be calculated as:

52 $$ C = \, 0. $$

With the vertical direction as positive, the vertical displacement equation *v* is:

53 $$ {\text{v}} = - \frac{{(3 + \mu )\rho \omega^{2} }}{E}\left( {ax^{2} sin\left( {b\left( {x - c} \right)} \right) - \frac{{\left( {2asin\left( {b\left( {x - c} \right)} \right) - 2abxcos\left( {b\left( {x - c} \right)} \right)} \right)}}{{b^{2} }}} \right). $$

### Finite element calculation of the MCD under centrifugal load

Since MCD is an axisymmetric structural unit, the axisymmetric finite element analysis method is applicable. When creating the finite element model, only a single section of the MCD needs to be established, greatly reducing the resource usage during analysis and improving the efficiency of analysis; the impact on the accuracy of the analysis is insignificant. The finite element model of MCD is established by the axisymmetric analysis method; only a single section of MCD is established. The volume element type is Solid185. Considering the model structure and calculation accuracy requirements, many sections are established on the model before grid division. Hence, hexahedral elements can be used for grid division. Elastic modulus, Poisson's ratio, and material density were set, the grid was divided into Smart Size (10), the analysis model was initially divided, and the local cell grid was refined; more than 80,000 cells were obtained^4^.

**Figure 7:**
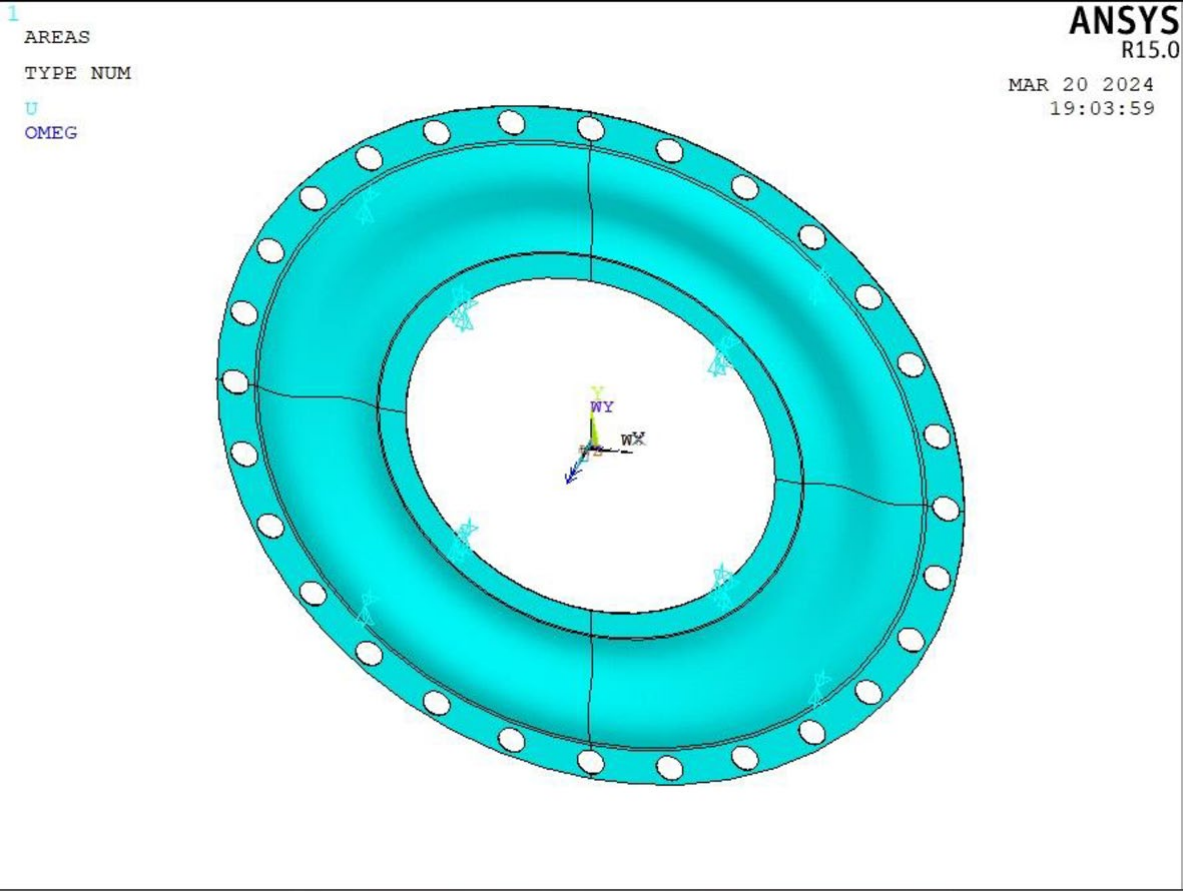
Finite element model of the MCD.

The finite element model of the MCD is established in ANSYS software, as shown in Fig. 7. All degrees of freedom are constrained at the inner hole spline, and the angular velocity around the Y-axis is applied. The structural and load parameters of the MCD are substituted to obtain a solution. Then, the circumferential force and deformation displacement of the waveform diaphragm under centrifugal load can be obtained.

## Results and discussion

### Comparative analysis of circumferential force results under centrifugal load

**Figure 8:**
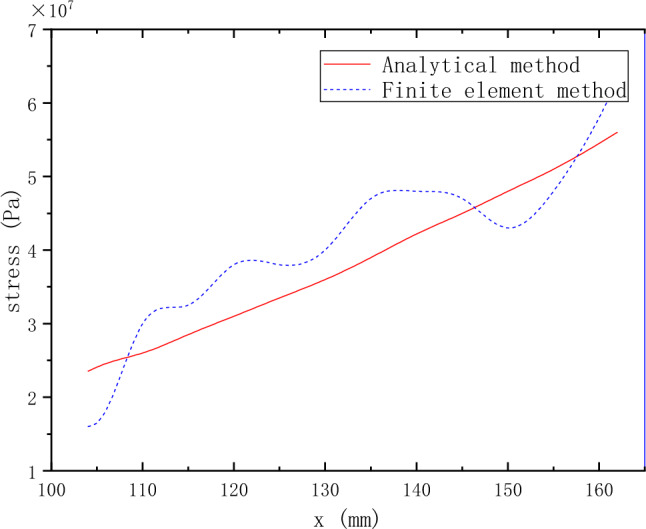
The circumferential stress curve obtained by analytical solution and the finite element method.

According to the above analysis method of force analysis of the corrugated film disk, the curve of the circular force $N_{\theta }$ relative to x of the MCD under the centrifugal force load can be obtained via MATLAB. The circumferential stress curves obtained by the analytical method and the finite element method are shown in Fig. 8.

A comparison of the circular stress obtained by the two methods reveals that the overall variation trend of the circular stress obtained by the analytical solution is the same as that obtained by ANSYS. However, the curve of the circular stress obtained by the analytical solution is smooth. In contrast, the curve of the circular stress obtained by ANSYS fluctuates. The maximum difference between the two methods is 19%.

### Comparative analysis of deformation displacement results under centrifugal load

**Figure 9:**
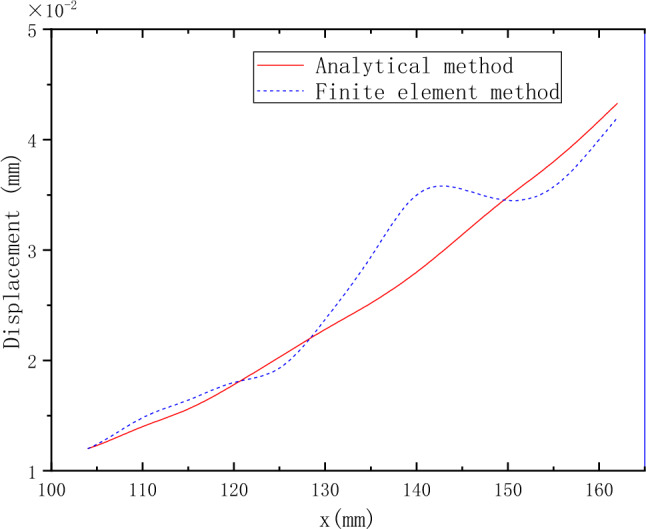
The horizontal displacement curve obtained by the analytical solution and the finite element method.

**Figure 10:**
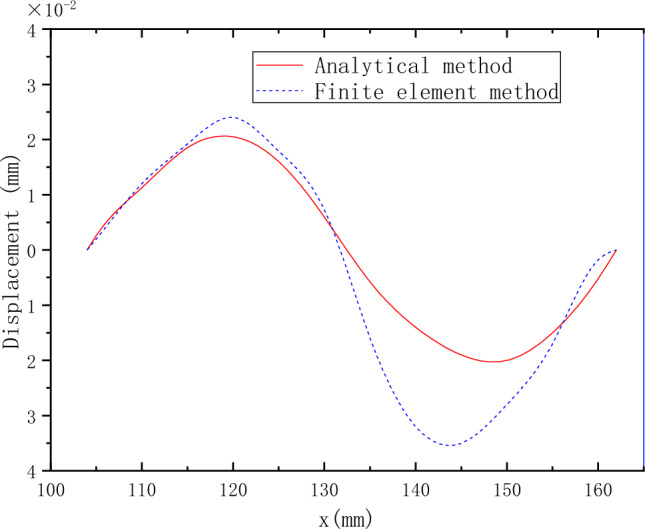
The vertical displacement curve obtained by the analytical solution and the finite element method.

The horizontal displacement *u* and vertical displacement *v* relative to x of the corrugated film disk under centrifugal force load can be obtained via MATLAB based on the above-conducted force analysis of the MCD. The horizontal and vertical displacement curves obtained by analytical and finite element methods are shown in Figs. 9 and 10, respectively.

The overall trend of horizontal displacement obtained by the analytical solution is the same as that obtained by ANSYS, and the overall value is also small. The maximum difference between the results obtained by the two methods is 19%. However, the horizontal displacement of the analytical solution gently increases while the result obtained by ANSYS fluctuates and increases. Secondly, the overall change trend of the vertical displacement obtained by the analytical solution is the same as that obtained by ANSYS. However, the overall value is small. The maximum difference between the two methods is 37%.

The results obtained by the finite element method have certain fluctuations in both stress and displacement. According to the further analysis of the MCD profile curve, when the profile radius is 118.5 mm, the MCD profile is at the peak position, where the stress and displacement fluctuate upward relative to the analytical solution. When the profile radius is 147.5 mm, the profile is at the trough position, where the stress and displacement fluctuate downward relative to the analytical solution. The difference between the analytical solution and the finite element results is closely related to the wavy film disk profile. The model is simplified, and the tangential force under the centrifugal force is neglected when the analytical solution is calculated. Under the centrifugal force load, the MCD produces additional deformation under the action of internal stress due to the special profile structure of the MCD. Consequently, tangential stress appears, leading to a fluctuation of the stress and displacement of the MCD relative to the theoretical value. However, because the mechanism of internal stress generation is relatively complicated, the analytical solution cannot be solved if the influence of tangential force generated by internal stress is considered. Therefore, optimizing the model cannot eliminate the fluctuation difference between the two results in this paper. However, according to refs.^1–5^, the stress generated by the MCD under centrifugal load is relatively small compared with that generated by other working conditions. In contrast, the maximum stress is only 6 MPa.

In summary, although there are some differences between the analytical and the finite element methods, introducing the rotating shell film model into the theoretical analysis of the MCD is still valid, providing a new way for the theoretical analysis of the MCD.

## Conclusions

This paper used the rotating shell thin film model to derive the corresponding relationship between the circumferential force and deformation displacement of the MCD and the centrifugal load. Then, the finite element method was used to analyze the circumferential force and deformation displacement of the MCD under the centrifugal load. The results obtained by the two methods are within the error range, proving the feasibility of introducing the rotating shell thin film model into the theoretical analysis method of the MCD. Consequently, a new method for the theoretical analysis of the MCD coupling was provided.

Although the results of the two methods in this paper are consistent within the error range, the error is still too large. In the future, a coupling stress test bench will be built, the stress test data will be combined, the analytical solution will be optimized to solve the equation and the finite element analysis model, and the results obtained by the two analysis methods will be reduced without affecting the force analysis. Thus, the development and application of MCD coupling will be promoted.

## Data Availability

The data supporting this study’s findings are available from the corresponding author upon reasonable request.
